# Bridging taxonomic gaps in microbial community profiling with LSTM-generated synthetic full-length 16S rRNA sequences

**DOI:** 10.1093/ismeco/ycag112

**Published:** 2026-04-23

**Authors:** Javier Geijo-Fernández, Alexander Pfundner, Carlos A Garcia-Perez

**Affiliations:** Division of Computational Systems Biology, University of Vienna, Universitätsring, 1, 1010 Vienna, Austria; Doctoral School in Microbiology and Environmental Science, University of Vienna, Universitätsring, 1, 1010 Vienna, Austria; Division of Computational Systems Biology, University of Vienna, Universitätsring, 1, 1010 Vienna, Austria; Doctoral School in Microbiology and Environmental Science, University of Vienna, Universitätsring, 1, 1010 Vienna, Austria; Digitalization & Transformation, Helmholtz Munich, Ingolstädter Landstraße, 1, 85764 Bavaria, Germany

**Keywords:** 16S rRNA, LSTM-based synthetic sequences, full-length amplicon profiling

## Abstract

Microbial community profiling relies on comprehensive reference databases, yet full-length 16S rRNA amplicons remain sparse for many bacterial taxa. We present SGenerator, a neural network-based data augmentation method that generates biologically informative, full-length (1500 bp) 16S rRNA sequences for underrepresented genera. Combining time series forecasting and natural language processing, SGenerator uses a long short-term memory (LSTM) architecture with a sliding-window approach and n-gram segmentation to generate full-length amplicons. Trained on a subset of 2289 sequences from 50 different unbalanced genera of a total of 184 732 high-quality sequences from the RiboGrove database, it produced 500 synthetic sequences per genus across 50 genera. BLASTn validation showed that an average of 300 sequences per genus closely matched native entries, and R2DT analysis confirmed that an average of 244 per genus folded into canonical 16S rRNA secondary structures, indicating strong biological fidelity. Classifiers trained on the augmented datasets achieved F1 and MCC scores of 0.90 on ITGDB and 0.75 on the more specialized MiDAS dataset, with k-mer embeddings slightly outperforming transformer-based representations. These results demonstrate that LSTM-driven sequence generation can effectively fill taxonomic gaps in full-length amplicon databases, overcome hypervariable region biases in short-read data, and has the potential to enhance microbial profiling accuracy in ecological studies.

## Introduction

Data generation has emerged as a demanding area of research in machine learning. Through data generation, the growing need for high-quality, diverse, and most importantly, realistic data that closely represents real-world data in datasets is addressed to enhance model performance.

The review by [1] summarizes methods for generating synthetic data and how machine learning models improve their performance. The review focuses on healthcare, privacy, and the sensitivity of real data, demonstrating how synthetic medical data can be used as an alternative to real patient data. For example, Neural Prophet [2] (a reviewed method) was used to generate synthetic data for temperature and glucose levels as time series data for patients with a diabetic foot. The results showed that using synthetic data to train models for diagnosing diabetes patients achieved accuracy levels as high as models trained only with real patient data. Other interesting models reviewed by [1] include SynSigGAN [3], GenerativeMTD [4], PepCVAE [5], SMOOTH-GAN [6], and [7], among others.

Long short-term memory (LSTM) networks [8] are a powerful tool for generating synthetic time series data, particularly when working with small datasets. LSTMs can model complex temporal patterns; e.g. GenerativeMTD and PepCVAE employ VAE-GAN-like architectures to address these challenges by focusing on augmenting small datasets while preserving statistical properties. A VAE-GAN architecture is the combination of variational autoencoders (VAE) with generative adversarial networks (GAN) [9]. Additionally, SynSigGAN has demonstrated how LSTM-based approaches can produce realistic synthetic data from limited real-world data, making them valuable for applications in fields where data is scarce or sensitive.

A notable application of LSTM to time series analysis is forecasting, commonly used for predicting natural phenomena or financial market trends. For example [10], explored a hybrid model that combined LSTM with multi-head attention mechanisms to improve forecasting accuracy. Despite using datasets as small as 43 samples, the model successfully predicted series that closely resembled real-world data, demonstrating the potential of LSTMs for effectively handling sequence-based tasks. Similarly [11], provided a comprehensive review of deep learning applications that applied LSTM to time series forecasting. The survey emphasized the effectiveness of LSTMs in sequence forecasting tasks, particularly due to their ability to capture long-term dependencies in sequential data.

### Generative modeling of sequences

Beyond their traditional application to time series forecasting, LSTMs have proven to be highly useful in the field of bioinformatics, particularly for generating biological sequences such as genes, genomes, proteins, and short motifs. These tasks demand the ability to model long-term dependencies and capture intricate patterns within biological data, making LSTMs an ideal choice.

In the study by [12], the authors developed a deep learning framework to predict non-coding DNA enhancer sequences involved in gene regulation. Due to the limited size of the dataset, they employed the RankGAN model [13] to augment the data. To generate sequences, the framework introduced a word embedding method, treating DNA sequences similarly to natural language phrases using word segmentation techniques commonly applied in Natural Language Processing tasks (NLP). For instance, the sequences were processed by sliding a window of three nucleotides, resulting in “words” such as AGA, CGT, and AGT until the end of the sequence. After augmenting the dataset, the deep learning framework achieved high accuracy in classifying enhancer sequences. Similarly, PERPHECT [14] is a computational framework designed to genetically engineer bacteriophages to enhance their host range. It includes a phage-bacterium interaction predictor [1D-convolutional neural network (CNN)] and a phage genome-sequence generator (LSTM). The generator was trained on genomic subsequences of length $n$, where a seed length is used to predict the next nucleotide in the sequence. Additionally, the generator was designed to predict phage genome edits to improve infection capabilities.

## SGenerator

Inspired by the studies described in the above sections, we based our approach on LSTM models, applying a combination of time series prediction techniques, as seen in [15]. The authors explained how to construct a training set from a single time series using a sliding window approach. In this method, the training set was created by splitting the original time series into overlapping subsequences of a fixed length $l$, referred to as the lag parameter. Each subsequence served as an input, with the subsequent value in the series as the output. For instance, given a sequence $T = [t_{1}, t_{2}, t_{3}, \ldots , t_{N}]$, the training pairs included inputs such as $[t_{1}, t_{2}, t_{3}]$ with corresponding outputs like $[t_{4}]$. After training, the LSTM model was able to forecast future values by leveraging patterns and dependencies learned from historical data. This approach ensured that the model learned to predict the next value in the series based on previous observations, effectively capturing the sequential nature of the data.

By implementing this technique, we were able to continue the sequence prediction over time, effectively lengthening the sequence to the desired length. In our study, we explore the predictive power of LSTMs on conserved nucleotide sequences by taking into account the previous $N$ base pairs to predict the next base. Additionally, as done in [12] and [14], we applied NLP techniques by segmenting sequences into chunks of size $n$, allowing the model to generate sequences from a random seed. Finally, to measure similarity to real sequences, we aligned the synthetic sequences using BLASTn as in [16]. Furthermore, the 16S rRNA is a highly conserved gene. To ensure that the generated sequences reached a minimum level of quality, we predicted their secondary structures to assess biological feasibility using the R2DT tool [17]. Our assumption was that a generated 16S sequence with a minimum level of intrinsic biological information should be able to fold. This quality control step ensured that we were not merely generating random sequences without biological value. As a result, our data augmentation approach can increase the number of sequences based on underrepresented taxonomic groups from a full-length amplicon database [18]. To our knowledge, no other study has augmented this type of data.

## Materials and methods

The following section outlines the methodology used to generate synthetic 16S sequences for dataset balancing prior to training a genus-level taxonomic classifier. The first part describes the pipeline for training the SGenerator models, including the analysis and selection criteria of the generated sequences. The second part details the evaluation of classifiers trained on augmented datasets.

The SGenerator and evaluation classifiers were implemented using PyTorch version 2.3.1 [19] and Scikit-Learn version 1.3.0 [20]. In addition, our Python code made use of Numpy version 1.26.4 [21], Pandas version 2.2.3 [22], and Seaborn version 0.13.2 [23]. We trained the model using CUDA version 12.1 [24] on an A100 GPU.

### Dataset for the SGenerator

We used the database RiboGrove v13.219 [18] to train the SGenerator models, and the ITGDB [25] and MiDAS [26] databases to validate the performance of classifiers trained from augmented datasets.

RiboGrove and MiDAS are two databases containing full-length 16S rRNA sequences, with RiboGrove aiming to represent the entire bacterial domain. On the one hand, RiboGrove is an optimal data source for training the SGenerator. On the other hand, MiDAS is a very specialized database focused only on wastewater treatment systems and anaerobic digesters, and thus serves as an ideal testing set for the classifier model due to its uniqueness. Finally, ITGDB is a known database of common short read sequences and can be used as general-purpose database to test the classifier across a wide spectrum of genera in contrast to the hyper-focused MIDAS database. The RiboGrove v13.219 database has a total of 185 654 sequences; after filtering the database by removing genus level sequences of “category 3” and sequences with “NA” genus, 184 732 sequences remained.

As a use case, we created a dataset with 50 different unbalanced genera to apply SGenerator and balance the subset with synthetic sequences. The selected subset from the RiboGrove database contains a total of 2289 sequences, where 9 genera consist of 100 to 300 sequences, and 41 genera consist of 20 to 50 sequences. The first column in Supplementary Table S1 shows the number of sequence samples per genus in the subset, and the second column shows the number of sequences that are required to balance the subset.

### Balanced dataset by the SGenerator

As shown in Supplementary Table S1, the genus that has the highest number of sequences is *Comamonas* with 222 sample sequences; thus, the dataset was balanced to 222 sequences per genus. The steps to apply the SGenerator were as follows.

**Figure 1:**
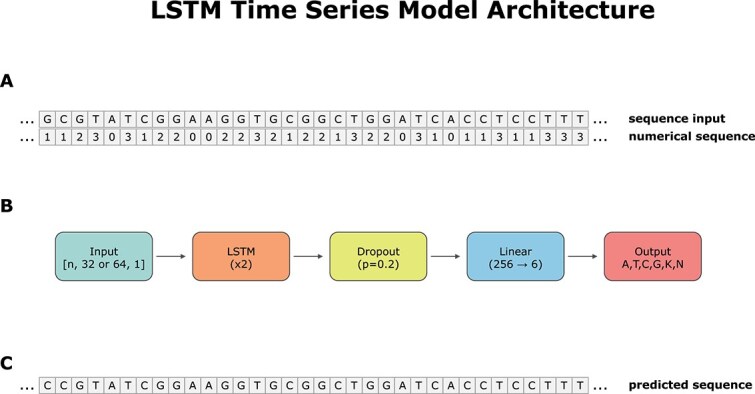
SGenerator model architecture. (A) The input string is transformed into a numerical representation to enable the LSTM network to learn time series patterns. (B) Overview of the SGenerator model architecture. (C) Output sequence generated based on the input seed sequence.

Since each genus sequence had its own feature characteristics, it was difficult to define an all-purpose architecture; however, we successfully defined three general architecture models for the 50 genera (see Fig. 1B for a summary of the architectures). All architecture models had three layers: an LSTM layer, a dropout layer, and a fully connected layer to predict the next nucleotide in the sequence. The difference between the architecture models lies in the number of recurrent layers and the number of nodes in the hidden layers. Specifically, Models 1 and 2 had 256 nodes in the hidden layers, while Model 3 had 512; Model 1 and Model 3 had two recurrent layers, whereas Model 2 had three recurrent layers. Supplementary Table S2 shows which model was used for each genus to generate synthetic sequences.

Initially, we used the Model 1 architecture (Supplementary Table S2) for all genera. Not all taxonomic generator models performed well; in other words, the generated sequences did not align with the training sequences using BLASTn. We increased the number of nucleotides (window size) to learn a range between 32 and 128 nucleotides. However, there were no improvements in the generated sequences for 9 genera. Therefore, the next step was to define a deeper architecture by adding another layer to the model and resetting the window size to test between 32 and 64 nucleotides. Afterwards, we retrained the SGenerator using Model 2 (Supplementary Table S2) only for those genera that did not perform well with the Model 1 architecture. We then ran a second BLASTn alignment to evaluate the Model 2 performance, in which 7 of the 9 genera aligned with the training sequences. Finally, for the remaining genera that failed to align, we reset the number of nucleotides to learn to 32 and increased the number of nodes in the architecture (Model 3, Supplementary Table S2), which improved the model performance; that is, the generated sequences aligned with the training sequences. Each genus was ultimately trained using either Model 1, Model 2, or Model 3.

### Sequence pre-processing

In order to train the SGenerator, we first had to separate the sequences by genus into individual FASTA files, resulting in 50 FASTA files used to train the models.

The pre-processing of the sequence FASTA files was performed as follows (Fig. 1). These steps were applied to each FASTA file:

1. We defined a vocabulary of DNA bases $(A, C, G, T, K, N)$ and mapped them to numerical values: $[A:0, C:1, G:2, T:3, K:4, N:5]$. $K$ and $N$ were included because they were present in the RiboGrove database.
2. We transformed each nucleotide in the sequence into a numerical sequence using the previously defined vocabulary (see Fig. 1A).
3. We obtained the maximum length of the sequences in the FASTA file.
4. We padded the sequences with zeros to match the maximum length.

### Training dataset processing and hyperparameters

After the pre-processing of the sequences, the creation of the training set for each SGenerator model proceeded as follows:

1. We set a window size of features to be learned by the model in order to predict the next feature (nucleotide) in the sequence. The window size depended on the selected model architecture and the genus, and was either 32 or 64 (see Supplementary Table S2).
2. For each numerical sequence, we extracted short sequences of a fixed window size (starting with a window size of 32) by shifting one position at a time until the end of the sequence was reached. We used the same short sequence as both input sample and label.
3. Afterwards, we reshaped the input data for the LSTM model into a vector (samples, time steps, features) and transformed the input data into PyTorch tensors, where samples corresponded to the total number of short sequences, time steps corresponded to the window size (32 or 64), and features equaled one, since we predicted the next nucleotide in the sequence.
4. We then transformed the input labels into PyTorch tensors.

The hyperparameters for training were as follows:

- We trained all models with 500 epochs.
- We set a batch size of 128, using the DataLoader function from PyTorch with data shuffling.
- We set the learning rate to the default value in PyTorch.
- We used Adam as the optimizer with the default values.
- We set the cross-entropy loss function with the default values.

Finally, we saved the best-performing model with the lowest loss.

### Generation of synthetic sequences

For each genus, 500 sequences were generated. First, we used a random short numerical seed sequence of the window size (32 or 64) as input to predict the next nucleotide. We then added the predicted nucleotide to the seed sequence and shifted one position forward to predict the next nucleotide. This process was repeated until the synthetic sequence reached the desired length. Second, we transformed the predicted numerical sequences into nucleotide sequences using the DNA vocabulary. Figure 1C shows an example of a synthetic sequence generated with SGenerator.

### Selection of synthetic sequences

The third column in Table 1 shows how many sequences are required to balance the dataset and train the classifier in our use case. To select which synthetic sequences were going to balance the dataset, we applied two methods to validate the generated sequences. The two methods provided are called quality and high-quality sequences.

**Table 1:** Total number of sequences required to balance the training set for the classifier.

Genus	Total sequence samples	Synthetic sequences to generate
*Acetivibrio*	29	193
*Alkaliphilus*	26	196
*Azoarcus*	25	197
*Cereibacter*	25	197
*Comamonas*	222	0
*Desulfosporosinus*	28	194
*Geobacter*	25	197
*Gordonia*	142	80
*Hydrogenophaga*	26	196
*Mesorhizobium*	122	100
*Microbacterium*	172	50
*Nocardioides*	110	112
*Paracoccus*	117	105
*Phascolarctobacterium*	25	197
*Pseudochrobactrum*	25	197
*Pseudoxanthomonas*	25	197
*Rhodanobacter*	26	196
*Rhodoferax*	29	193
*Rhodopseudomonas*	30	192
*Roseburia*	29	193
*Ruminococcus*	25	197
*Selenomonas*	27	195
*Sphingobium*	117	105
*Sphingomonas*	147	75
*Sulfurospirillum*	30	192
*Thermoanaerobacterium*	25	197
*Thiopseudomonas*	26	196
*Treponema*	152	70

### Quality sequences

The quality sequences relied on applying BLASTn [27] to the training FASTA files and the synthetic sequences generated by SGenerator, which were saved as FASTA files.

For each FASTA file, we ran a local BLASTn with the following parameters:

1. Each synthetic sequence in the FASTA file was used as a query.
2. Each sequence in the FASTA file used to train the SGenerator was used as the subject.
3. The BLASTn output was formatted as a table with the following columns: query, subject, percentage of identity, length of alignment, subject length, query position start, query position end, subject position start, subject position end, and e-value.
4. The BLASTn results included only sequences with an e-value of at most 1e-5, which is the default BLASTn threshold.

We filtered the BLASTn results as follows: We selected the synthetic sequences with a length equal to or greater than 1000 nucleotides that aligned with the training sequences. We then created a filtered FASTA file containing sequences longer than 1000 nucleotides with an e-value of at most 1e-5.

### High-quality sequences

**Figure 2:**
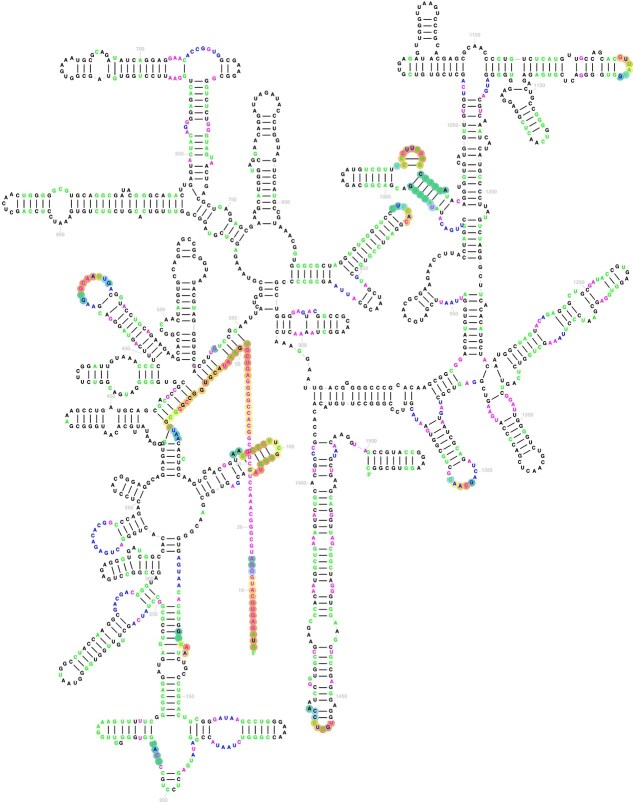
Generated 16S RNA sequence folded by R2DT, with colours indicating nucleotide identity relative to the template: identical nucleotides in black, insertions in magenta, modifications in green, and repositioned nucleotides in blue.

The generation of the high-quality sequences included one additional step following the creation of the filtered FASTA file described above. After filtering the BLASTn results, we predicted the secondary structure of the generated RNA sequences using the R2DT tool [17] (see Fig. 2). The balanced dataset generated with our method contained both quality and high-quality sequences. In some genera, not all synthetic samples were high-quality sequences (see Supplementary Table S3).

As an additional step, we explored the biological contribution of the synthetic sequences by running a phylogenetic analysis. To compute the phylogenetic tree, we selected 10 random synthetic sequences and 10 random sequences from the MiDAS database for each of the 50 genera. Due to the large size of the resulting tree, we did not show it in the main manuscript; however, it was provided as Supplementary Material.

We embedded and visualized real and synthetic sequences using principal component analysis (PCA) and t-distributed stochastic neighbor embeddingt-SNE) projections with points colored by data origin. Per-genus projections are provided to enable a finer-grained assessment of whether synthetic sequences follow the same distribution as real sequences within each genus (Supplementary Figs S3, S4, S5, and S6)

### Assessment of classifier effectiveness on augmented datasets

To assess the utility of synthetic sequences generated by SGenerator for genus-level taxonomic classification, we constructed a mixed dataset consisting of both real 16S rRNA sequences from RiboGrove and synthetic sequences generated per genus. The resulting mixed dataset, comprising 8023 sequences in total, included 1807 real sequences and 6216 synthetic sequences, and was used for both model training and evaluation. In addition to the evaluation on a held-out test set, the trained models were further assessed on external datasets to evaluate their generalization performance.

#### Sequence pre-processing and vectorization

All 16S rRNA sequences were first converted into numerical representations suitable for machine learning models using two different embedding strategies:

- k-mer frequency embedding: Sequences were decomposed into all possible overlapping k-mers of length 3 using an alphabet consisting of $A, C, G, T, K, \textrm{ and } N$. The frequency of each of the 216 possible k-mers was computed, resulting in a fixed-length feature vector representing k-mer composition.
- Transformer-based embedding: Sequences were tokenized and passed through a pretrained DNABERT-S model [28]. The transformer-based model produced hidden state vectors, which were mean-pooled across all tokens to yield a fixed-length embedding of 768 dimensions.

To verify the structure and separability of the resulting embeddings, we performed visual inspection using ordination techniques, like PCA and t-SNE.

#### Train/test split strategy

To rigorously test model generalization and reduce overfitting to highly similar sequences, we applied a group split strategy utilizing GroupShuffleSplit from scikit-learn [20]. Groups were defined by clustering sequences at 95% identity using VSEARCH 2.30.0 [29], ensuring that sequences from the same cluster do not appear in both training and testing sets. The dataset was split into 80% for training and 20% for testing, resulting in a training set of 6444 sequences and a test set of 1579 sequences.

#### Classifier modeling

For each embedding type, we trained five classifiers, including traditional machine learning models such as random forest (RF), support vector machine (SVM), and XGBoost [30], as well as shallow neural network architectures including a feedforward neural network (FNN) and a CNN. First, RF, SVM, and XGBoost were trained using four-fold cross-validation on the training split. Then, they were retrained on the full training set and evaluated on the test set using default parameters. FNN and CNN were implemented in PyTorch [19]. The FNN consisted of two hidden layers with 64 and 128 units and ReLU activations. The CNN included two one-dimensional convolutional layers with 64 filters of size 5 and 128 filters of size 3, followed by a fully connected layer. Both models were trained with early stopping and a learning rate scheduler to prevent overfitting.

#### Evaluation on external datasets

To assess model robustness, the final models were evaluated using two external datasets. The first, MiDAS [26], is a domain-specific, full-length 16S dataset derived from wastewater environments. The second, ITGDB [25], is a more general-purpose 16S rRNA sequence database based on common short-read amplicons. These external datasets were used to evaluate the transferability of models trained on augmented datasets.

## Results

### SGenerator: synthetic sequence generator

Supplementary Table S3 shows the results of the SGenerator. In columns three and four, the total number of sequences with a BLASTn match and the fraction of those sequences with predicted secondary structures from the R2DT tool are shown.

A total of 500 sequences were generated for each genus. As shown in Supplementary Table S3, a high proportion of the generated sequences have predicted secondary structures and can be used to balance the dataset. On average, 300 generated sequences per genus had a BLASTn hit against the RiboGrove database, and 244 sequences had predicted secondary structures. Nevertheless, not all generated sequences were able to fold, such as those from the following genera: *Cellulosilyticum* (195), *Sulfurovum* (135), *Shinella* (150), *Pseudochrobactrum* (172), *Rhodoferax* (186), *Paracoccus* (92), and *Treponema* (66).

For genera with more than 300 high-quality generated sequences, we randomly selected sequences to balance the sample classes. For genera in which not all generated sequences were of high quality, we selected sequences from the BLASTn hits to balance the samples. All generated sequences can be found in the (GitLab project: https://gitlab.com/charlos1204/sgenerator.git).

**Figure 3:**
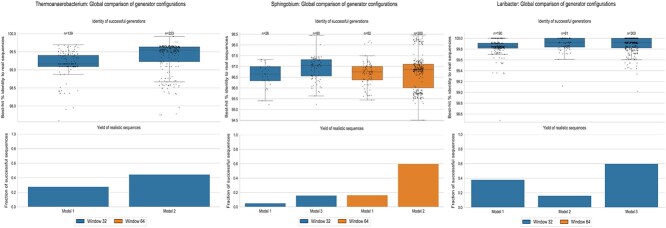
Comparison of SGenerator model configurations across three representative genera (*Thermoanaerobacterium*, *Sphingobium*, and *Laribacter*). Top row: Boxplots showing the distribution of best-hit BLASTn percent identity between synthetic and real sequences. Dots represent individual synthetic sequences, and boxplots summarize the median and interquartile range. Model 2 is absent for *Sphingobium* because it did not produce sequences meeting BLASTn significance or length criteria. Bottom row: Bar plots showing the yield of synthetic sequences, defined as the fraction of generated sequences with a significant BLASTn hit. The plots highlight differences in sequence identity and generation efficiency across models and genera.

For each model configuration, 500 synthetic sequences were generated per genus. Model performance was evaluated in terms of both sequence quality and yield, where quality was defined as the best-hit percentage identity to real sequences and yield as the fraction of generated sequences producing a significant BLASTn match. Figure 3 shows representative results for three genera (*Thermoanaerobacterium*, *Sphingobium*, and *Laribacter*) across the three SGenerator models. The boxplots (Fig. 3, top) indicate that all models generate sequences with high identity to real genomes, with Models 2 and 3 generally achieving higher median identity, although with increased variability for some genera. In contrast, the bar plots (Fig. 3, bottom) show marked differences in yield between models, with Model 1 consistently producing fewer successful sequences and Models 2 and 3 substantially improving generation efficiency, particularly for *Laribacter*.

For the genus *Thermoanaerobacterium*, a window size of 32 using Model 2 reached the required 197 sequences to balance the dataset for that class, making it unnecessary to scale to a more complex architecture. In contrast, *Sphingobium* required increasing the window size to 64 when using Model 2, as a window size of 32 did not produce a sufficient number of quality sequences; moreover, Model 2 produced no BLASTn hits for this genus, as shown in Fig. 3. Finally, for *Laribacter*, Model 3 with a window size of 32 was able to generate 300 quality sequences, demonstrating the benefit of architectural scaling. Results for the remaining genera using different model and window-size combinations are reported in the Supplementary Material.

### Phylogenetic tree analysis

Supplementary Fig. S9 shows a subtree extracted from the phylogenetic tree, which was constructed using the MiDAS test database together with the synthetic sequences generated by SGenerator. The subtree contains sequences from the *Gordonia* and *Dietzia* genera, where real sequences are shown in blue and synthetic sequences in orange. The SGenerator model for the genus *Dietzia* was trained using 20 sequences from the RiboGrove database, whereas the model for *Gordonia* was trained with 142 sequences. Synthetic sequences were generated using Model 1 with a window size of 32. It is clear that the synthetic *Gordonia* sequences exhibit shorter branch lengths and are more closely related to the real sequences, likely due to the larger number of training samples available. In contrast, the synthetic *Dietzia* sequences show longer branch lengths, indicating greater divergence from the real sequences. Nevertheless, these results indicate that Model 1 successfully learned the key features required to generate sequences highly similar to real ones even from a limited number of training samples, as all ten synthetic sequences cluster within the same branch as the real sequences.

The subtree shown in Supplementary Fig. S11 includes four genera: *Acetivibrio*, *Ruminococcus*, *Ruminiclostridium*, and *Thermoanaerobacterium*. It is important to note that all four genera were trained using between 21 and 29 sequences and a window size of 32. Notably, the genus *Thermoanaerobacterium* was trained using Model 2, whereas the remaining genera used Model 1. Specifically, for the genus *Thermoanaerobacterium*, all synthetic sequences are located within the main branch of the real sequences, while some real sequences also cluster within a sub-branch predominantly composed of synthetic sequences. This observation shows an improvement in sequence generation achieved by scaling the SGenerator architecture.

In Supplementary Fig. S12, the subtree includes four genera: *Shinella*, *Methylocystis*, *Mesorhizobium*, and *Pseudochrobactrum*. The genera *Shinella* and *Methylocystis* were trained using Model 1 with a window size of 32 and contain 21 and 24 sequences, respectively. In contrast, the genus *Mesorhizobium* was trained using Model 2 with a window size of 64 and 122 sequences, while *Pseudochrobactrum* was trained using Model 3 with a window size of 32 and it includes 25 sequences.

For the genera *Shinella* and *Pseudochrobactrum*, the tree shows a hierarchical clustering pattern in which the *Shinella* branch constitutes the parent clade for both real and synthetic sequences of the two genera. Specifically, while the real *Shinella* sequences form a distinct sub-branch, the sub-branch dominated by synthetic *Shinella* sequences contains both real and synthetic sequences from *Pseudochrobactrum*. In contrast, for the genus *Mesorhizobium*, the real and synthetic sequences are grouped into well-defined and genus-consistent sub-branches, which can be attributed to the larger number of training sequences and the model architecture used to generate the synthetic data.

In Supplementary Fig. S10, the subtree corresponding to the genus *Laribacter* shows a correct clustering of synthetic sequences with the real ones. It is worth noting that Model 3 was used to generate the synthetic sequences; however, unlike the genus *Mesorhizobium*, the model was trained using only 21 sequences. This result suggests that Model 3 is able to capture robust sequence patterns even from a limited number of training examples, supporting the biological relevance of the synthetic sequences generated by the SGenerator.

Overall, these observations suggest that, despite of being trained on a different dataset, the SGenerator produces synthetic sequences that preserve informative phylogenetic structure when evaluated on an independent database, even in cases showing complex clustering patterns such as *Shinella* and *Pseudochrobactrum*.

### Performance of classifiers trained on the augmented dataset

To evaluate the effectiveness of the learned embeddings and the trained classifiers, we assessed their performance across multiple evaluation settings. This included a holdout test set drawn from the same distribution as the training data, as well as two external datasets: ITGDB and MiDAS, which represent distinct microbial environments. These external datasets serve as a benchmark for testing model generalization. MiDAS contains longer sequences from wastewater treatment systems, while ITGDB consists of shorter sequences from the human gut microbiome. These biological and technical differences provide a meaningful test of the robustness of both the embeddings and the classification models.

#### Embedding structure and taxonomic signal

To assess whether the sequence embeddings captured taxonomic structure, we applied PCA and t-SNE to both the k-mer frequency and transformer-based (DNABERT-S) representations using scikit-learn implementations (Supplementary Figs S1 and S2). The resulting two-dimensional ordination plots showed that taxonomic structure was partially preserved in both embedding spaces. While a clear and complete separation between taxonomic groups was not observed, sequences belonging to the same genus appear often in proximity. No substantial difference in visual separability was noted between the two embedding approaches. In PCA, the first two components explained approximately 30% of the variance for the RiboGrove dataset and 20% for the mixed dataset, indicating that a modest portion of the embedding variation is captured in the low-dimensional space.

#### Classifier performance evaluation

Models achieved high performance across all datasets (Tables 2–5), with the highest metrics observed on the holdout test set, where accuracy, MCC, F1-score, precision, and recall all exceeded 0.95 for both embedding types. Performance was slightly lower on the ITGDB dataset, but remained within a high range across all metrics. On the more challenging MiDAS dataset, a decline in performance was observed as expected, reflecting its increased complexity due to longer sequences, though the models still achieved reasonable classification metrics. Across datasets, differences between k-mer and DNABERT-S embeddings were generally modest, with k-mer yielding marginally higher scores in most cases. We additionally report baseline results from classifiers trained exclusively on real sequences. On the holdout test set, these baseline models achieved, on average, $\sim $7% lower accuracy than models trained on augmented data (Supplementary Tables S4 and S5). In contrast, performance on external datasets is comparable between real-only and augmented models, indicating that the inclusion of synthetic sequences does not negatively affect the generalization with regard to independent datasets (Supplementary Tables S6 and S7).

**Table 2:** Performance on holdout test set using k-mer embedding.

Model	Accuracy	MCC	F1	Precision	Recall
SVM	0.92	0.91	0.92	0.94	0.92
RF	0.97	0.97	0.97	0.98	0.97
XGB	0.96	0.96	0.96	0.97	0.96
FNN	0.97	0.97	0.97	0.98	0.97
CNN	0.99	0.99	0.99	0.99	0.99
	**0.96**	**0.96**	**0.96**	**0.97**	**0.96**

Bold values indicate average performance across models.

**Table 3:** Performance on holdout test set using DNABERT-S embedding.

Model	Accuracy	MCC	F1	Precision	Recall
SVM	0.96	0.96	0.96	0.98	0.96
RF	0.93	0.92	0.93	0.95	0.93
XGB	0.92	0.92	0.93	0.95	0.92
FNN	0.96	0.96	0.97	0.98	0.96
CNN	0.98	0.97	0.98	0.98	0.98
	**0.95**	**0.95**	**0.95**	**0.97**	**0.95**

Bold values indicate average performance across models.

**Table 4:** Performance on external datasets using k-mer embedding.

	Model	Accuracy	MCC	F1	Precision	Recall
ITGDB	SVM	0.92	0.92	0.91	0.93	0.92
	RF	0.91	0.90	0.91	0.93	0.91
	XGB	0.83	0.82	0.84	0.88	0.83
	FNN	0.92	0.92	0.94	0.96	0.92
	CNN	0.95	0.94	0.95	0.96	0.95
		**0.91**	**0.90**	**0.91**	**0.93**	**0.91**
MiDAS	SVM	0.83	0.82	0.81	0.85	0.83
	RF	0.64	0.62	0.62	0.80	0.64
	XGB	0.57	0.55	0.58	0.75	0.57
	FNN	0.86	0.85	0.86	0.89	0.86
	CNN	0.91	0.90	0.90	0.93	0.91
		**0.76**	**0.75**	**0.75**	**0.84**	**0.76**

Bold values indicate average performance across models for each dataset.

**Table 5:** Performance on external datasets using DNABERT-S embedding.

	Model	Accuracy	MCC	F1	Precision	Recall
ITGDB	SVM	0.92	0.92	0.94	0.96	0.92
	RF	0.84	0.83	0.87	0.92	0.84
	XGB	0.80	0.79	0.82	0.87	0.80
	FNN	0.87	0.87	0.89	0.93	0.87
	CNN	0.81	0.80	0.86	0.93	0.81
		**0.85**	**0.84**	**0.88**	**0.92**	**0.85**
MiDAS	SVM	0.86	0.85	0.87	0.90	0.86
	RF	0.73	0.71	0.74	0.83	0.73
	XGB	0.70	0.68	0.71	0.77	0.70
	FNN	0.78	0.76	0.79	0.86	0.78
	CNN	0.69	0.67	0.71	0.87	0.69
		**0.75**	**0.74**	**0.76**	**0.85**	**0.75**

Bold values indicate average performance across models for each dataset.

## Discussion

As shown in the results section, most of the sequences generated by the SGenerator were able to fold above 75% for each of the genera with a few exceptions such as *Paracoccus* and *Treponema*. Overall, the model successfully produced high-quality sequences for the majority of genera. One possible reason for this strong performance is the low variation rate of the 16S rRNA gene. This property is one of the main reasons 16S is widely used to classify new bacteria. Additionally, with fewer variations in the sequence, LSTM models are more likely to learn the patterns effectively and to predict the next output accurately.

To prevent overfitting, we introduced dropout layers into the SGenerator’s architecture. The models’ design (see Supplementary Table S2) consisted of three different architectural variants. Model 1, the baseline, used a 32-nucleotide window and was able to learn the necessary features to predict the next sequence occurrence without over- or underfitting for most genera. However, for certain genera such as *Thermoanaerobacterium*, *Pseudoxanthomonas*, or *Sphingomonas* to mention a few, Model 1 began to underfit, resulting in decreased performance.

To address this, we first increased the window size from 32 to 64 nucleotides. However, the results remained unsatisfactory despite the adjustment. Next, we increased the number of nodes per layer from 256 to 512, which still did not yield improvements. Finally, we added a third layer with 256 nodes in each layer and used a mix of 32 and 64 nucleotide windows. This configuration led to performance improvements, forming the basis of Model 2, which required a deeper architecture to predict the next sequence occurrence accurately.

Model 3 was developed for genera such as *Laribacter* and *Pseudochrobactrum*, which continued to underfit even with Model 2. Based on our prior experience, we increased the number of nodes from 256 to 512 across the layers. This adjustment led to a notable improvement in performance. In other words, Model 3 required a wider network to make accurate predictions.

To ensure the biological relevance of the generated sequences, we applied quality filters such as BLASTn and R2DT. Specifically, we used BLASTn as an initial filter to retain only the sequences with high similarity, thereby increasing the likelihood that the sequences would successfully fold and possess biological significance.

Importantly, secondary structure folding alone was not treated as evidence that synthetic sequences fully recapitulate the true data manifold, but rather as an initial biological plausibility filter. Stronger support for biological relevance is provided by the preservation of genus-consistent phylogenetic structure when synthetic sequences are integrated with real sequences from an independent external 16S rRNA database, together with maintained generalization performance on external classification benchmarks.

While data augmentation can enhance model robustness and generalization by increasing the diversity of input sequences, it also carries the risk of introducing biases or synthetic patterns that may lead to overfitting if not biologically meaningful. To mitigate this, an evaluation based on independent and real-world datasets was emphasized to ensure that performance metrics reflect genuine predictive ability rather than artifacts of augmentation. Additionally, a balance must be maintained between expanding dataset size and preserving the biological relevance of augmented sequences.

Finally, our approach demonstrates that augmentation can enable effective modeling of rare taxa where the number of real sequences is inherently limited, supporting broader applicability in taxonomic classification tasks.

## Conclusion

The SGenerator has proven to have potential for generating biologically meaningful 16S rRNA synthetic sequences across a wide range of bacterial genera. Its success can be attributed to the architectural adaptability of the model and, to some extent, the low variability of the 16S gene. By iteratively adjusting window sizes, increasing the number of nodes, and deepening the network, we were able to address cases of underfitting and optimize performance for more challenging genera. Furthermore, the integration of biological validation tools such as BLASTn and R2DT ensured that the synthetic sequences not only performed well statistically but also maintained biological relevance (i.e., similarity). The results suggest that the SGenerator can serve as a reliable tool for synthetic sequence generation in microbial taxonomy and related bioinformatics applications.

Classifiers that were trained by using the proposed data representations demonstrated consistently high performance across both holdout and external datasets, indicating robust generalization. Performance remained stable even in more complex or taxonomically diverse datasets. Both embedding strategies were effective, while k-mer embeddings showed a slight overall advantage. These results support the validity of the approach for sequence-based classification tasks in microbial genomics.

Finally, as future work, we are confident in the performance of our method and intend to expand its applicability to other organisms, such as viral proteins. We anticipate that these extensions will solidify the role of synthetic sequence generation as a cornerstone technology in next-generation bioinformatics.

## Supplementary Material

Supplementary-Material_ycag112

## Data Availability

The data and code that support the findings of this study are publicly available in the GitLab repository at https://gitlab.com/charlos1204/sgenerator.git.
