# Supplementary material for: Bridging taxonomic gaps in microbial community profiling with LSTM-generated synthetic full-length 16S rRNA sequences
Source: ISME Commun. 2026 Apr 23;6(1):ycag112. doi: 10.1093/ismeco/ycag112 (PMC13196590; doi:10.1093/ismeco/ycag112)
Supplement: Supplementary-Material_ycag112 [file supplementary-material_ycag112.zip › Supplementary_Material_ycag112.pdf]

Supplementary Materials

2025

1 Tables

Table S1: Sub-dataset samples

Genus	Total sequence samples	Synthetic sequences to generate
Dietzia	20	202
Psychromonas	20	202
Dialister	20	202
Dyadobacter	20	202
Tessaracoccus	20	202
Laribacter	21	201
Methylocystis	21	201
Ruminiclostridium	21	201
Sulfurovum	22	200
Cellulosilyticum	22	200
Coprococcus	22	200
Coxiella	22	200
Sphingopyxis	22	200
Megamonas	23	199
Methanothermobacter	23	199
Methanobacterium	23	199
Secundilactobacillus	23	199
Pelosinus	23	199
Vitreoscilla	23	199
Dechloromonas	23	199
Anaerocolumna	24	198
Shinella	24	198
Cereibacter	25	197
Thermoanaerobacterium	25	197
Pseudochrobactrum	25	197
Phascolarctobacterium	25	197
Azoarcus	25	197
Geobacter	25	197
Pseudoxanthomonas	25	197
Ruminococcus	25	197
Rhodanobacter	26	196
Thiopseudomonas	26	196
Alkaliphilus	26	196
Hydrogenophaga	26	196
Selenomonas	27	195
Desulfosporosinus	28	194
Acetivibrio	29	193
Roseburia	29	193

Continued on next page

Genus	Total sequence samples	Synthetic sequences to generate
Rhodoferax	29	193
Rhodopseudomonas	30	192
Sulfurospirillum	30	192
Nocardioides	110	112
Paracoccus	117	105
Sphingobium	117	105
Mesorhizobium	122	100
Gordonia	142	80
Sphingomonas	147	75
Treponema	152	70
Microbacterium	172	50
Comamonas	222	0

Table S2: Summary of trained models per genus

Genus	Model architecture	Window size
Dietzia	Model 1	32
Psychromonas	Model 1	32
Dialister	Model 1	32
Dyadobacter	Model 1	32
Tessaracoccus	Model 1	32
Methylocystis	Model 1	32
Ruminiclostridium	Model 1	32
Sulfurovum	Model 1	32
Cellulosilyticum	Model 1	32
Coprococcus	Model 1	32
Coxiella	Model 1	32
Sphingopyxis	Model 1	32
Megamonas	Model 1	32
Methanothermobacter	Model 1	32
Methanobacterium	Model 1	32
Secundilactobacillus	Model 1	32
Pelosinus	Model 1	32
Vitreoscilla	Model 1	32
Dechloromonas	Model 1	32
Anaerocolumna	Model 1	32
Shinella	Model 1	32
Cereibacter	Model 1	32
Phascolarctobacterium	Model 1	32
Azoarcus	Model 1	32
Geobacter	Model 1	32
Ruminococcus	Model 1	32
Rhodanobacter	Model 1	32
Thiopseudomonas	Model 1	32
Hydrogenophaga	Model 1	32
Selenomonas	Model 1	32
Desulfosporosinus	Model 1	32
Acetivibrio	Model 1	32
Roseburia	Model 1	32
Rhodoferax	Model 1	32

Continued on next page

Genus	Model architecture	Window size
Rhodopseudomonas	Model 1	32
Sulfurospirillum	Model 1	32
Nocardioides	Model 1	32
Gordonia	Model 1	32
Microbacterium	Model 1	32
Comamonas	Model 1	32
Alkaliphilus	Model 1	32
Thermoanaerobacterium	Model 2	32
Pseudoxanthomonas	Model 2	32
Paracoccus	Model 2	64
Sphingobium	Model 2	64
Mesorhizobium	Model 2	64
Sphingomonas	Model 2	64
Treponema	Model 2	64
Laribacter	Model 3	32
Pseudochrobactrum	Model 3	32

Table S3: High quality sequences generated

Genus	Total sequences in database	Predicted match blast	Predicted folded	Need to predict
Dialister	20	250	250	202
Dietzia	20	243	240	202
Dyadobacter	20	345	280	202
Psychromonas	20	395	255	202
Tessaracoccus	20	481	228	202
Laribacter	21	407	328	201
Methylocystis	21	285	280	201
Ruminiclostridium	21	360	359	201
Cellulosilyticum	22	423	195	200
Coprococcus	22	291	289	200
Coxiella	22	246	246	200
Sphingopyxis	22	309	309	200
Sulfurovum	22	359	135	200
Dechloromonas	23	208	206	199
Megamonas	23	263	263	199
Methanobacterium	23	225	224	199
Methanothermobacter	23	411	312	199
Pelosinus	23	341	341	199
Secundilactobacillus	23	298	298	199
Vitreoscilla	23	462	219	199
Anaerocolumna	24	375	375	198
Shinella	24	227	150	198
Azoarcus	25	500	230	197
Cereibacter	25	257	248	197
Geobacter	25	211	208	197
Phascolarctobacterium	25	343	220	197
Pseudochrobactrum	25	404	172	197
Pseudoxanthomonas	25	234	231	197
Ruminococcus	25	222	216	197

Continued on next page

Genus	Total sequences in database	Predicted match blast	Predicted folded	Need to predict
Thermoanaerobacterium	25	205	205	197
Alkaliphilus	26	230	230	196
Hydrogenophaga	26	317	199	196
Rhodanobacter	26	248	248	196
Thiopseudomonas	26	244	238	196
Selenomonas	27	340	340	195
Desulfosporosinus	28	309	308	194
Acetivibrio	29	335	326	193
Rhodoferax	29	216	186	193
Roseburia	29	252	250	193
Rhodopseudomonas	30	266	265	192
Sulfurospirillum	30	453	208	192
Nocardioides	110	314	310	112
Paracoccus	117	184	92	105
Sphingobium	117	265	265	105
Mesorhizobium	122	138	130	100
Gordonia	142	410	340	80
Sphingomonas	147	226	137	75
Treponema	152	73	66	70
Microbacterium	172	414	399	50
Comamonas	222	193	191	0

Table S4: Baseline performance on the holdout test set using k-mer embeddings. Classifiers were trained solely on original (Ribogrove) sequences.

Model	Accuracy	MCC	F1	Precision	Recall
SVM	0.81	0.75	0.78	0.76	0.81
RF	0.93	0.90	0.92	0.93	0.93
XGB	0.83	0.77	0.83	0.90	0.83
FNN	0.94	0.92	0.96	0.99	0.94
CNN	0.96	0.95	0.98	1.00	0.96
	**0.89**	**0.86**	**0.89**	**0.92**	**0.89**

Table S5: Baseline performance on the holdout test set using DNABERT-S embeddings. Classifiers were trained solely on original (Ribogrove) sequences.

Model	Accuracy	MCC	F1	Precision	Recall
SVM	0.91	0.88	0.93	1.00	0.91
RF	0.84	0.79	0.84	0.93	0.84
XGB	0.84	0.79	0.86	0.98	0.84
FNN	0.90	0.87	0.94	0.99	0.90
CNN	0.90	0.87	0.92	0.98	0.90
	**0.88**	**0.84**	**0.90**	**0.98**	**0.88**

Table S6: Baseline performance on external datasets using k-mer embedding. Classifiers were trained solely on original (Ribogrove) sequences.

	Model	Accuracy	MCC	F1	Precision	Recall
ITGDB	SVM	0.92	0.92	0.91	0.93	0.92
	RF	0.91	0.90	0.91	0.93	0.91
	XGB	0.83	0.82	0.84	0.88	0.83
	FNN	0.91	0.91	0.93	0.96	0.91
	CNN	0.98	0.98	0.98	0.98	0.98
		**0.91**	**0.91**	**0.91**	**0.94**	**0.91**
MiDAS	SVM	0.83	0.82	0.81	0.85	0.83
	RF	0.64	0.62	0.62	0.80	0.64
	XGB	0.57	0.55	0.58	0.75	0.57
	FNN	0.86	0.85	0.87	0.89	0.86
	CNN	0.96	0.96	0.96	0.96	0.96
		**0.77**	**0.76**	**0.77**	**0.85**	**0.77**

Table S7: Baseline performance on external datasets using DNABERT-S embedding. Classifiers were trained solely on original (Ribogrove) sequences.

	Model	Accuracy	MCC	F1	Precision	Recall
ITGDB	SVM	0.92	0.92	0.94	0.96	0.92
	RF	0.84	0.83	0.87	0.92	0.84
	XGB	0.80	0.79	0.82	0.87	0.80
	FNN	0.82	0.81	0.83	0.87	0.82
	CNN	0.84	0.83	0.85	0.90	0.84
		**0.85**	**0.84**	**0.86**	**0.90**	**0.85**
MiDAS	SVM	0.86	0.85	0.87	0.90	0.86
	RF	0.73	0.71	0.74	0.83	0.73
	XGB	0.70	0.68	0.71	0.77	0.70
	FNN	0.75	0.73	0.75	0.82	0.75
	CNN	0.79	0.77	0.79	0.86	0.79
		**0.76**	**0.75**	**0.77**	**0.84**	**0.76**

2 Figures

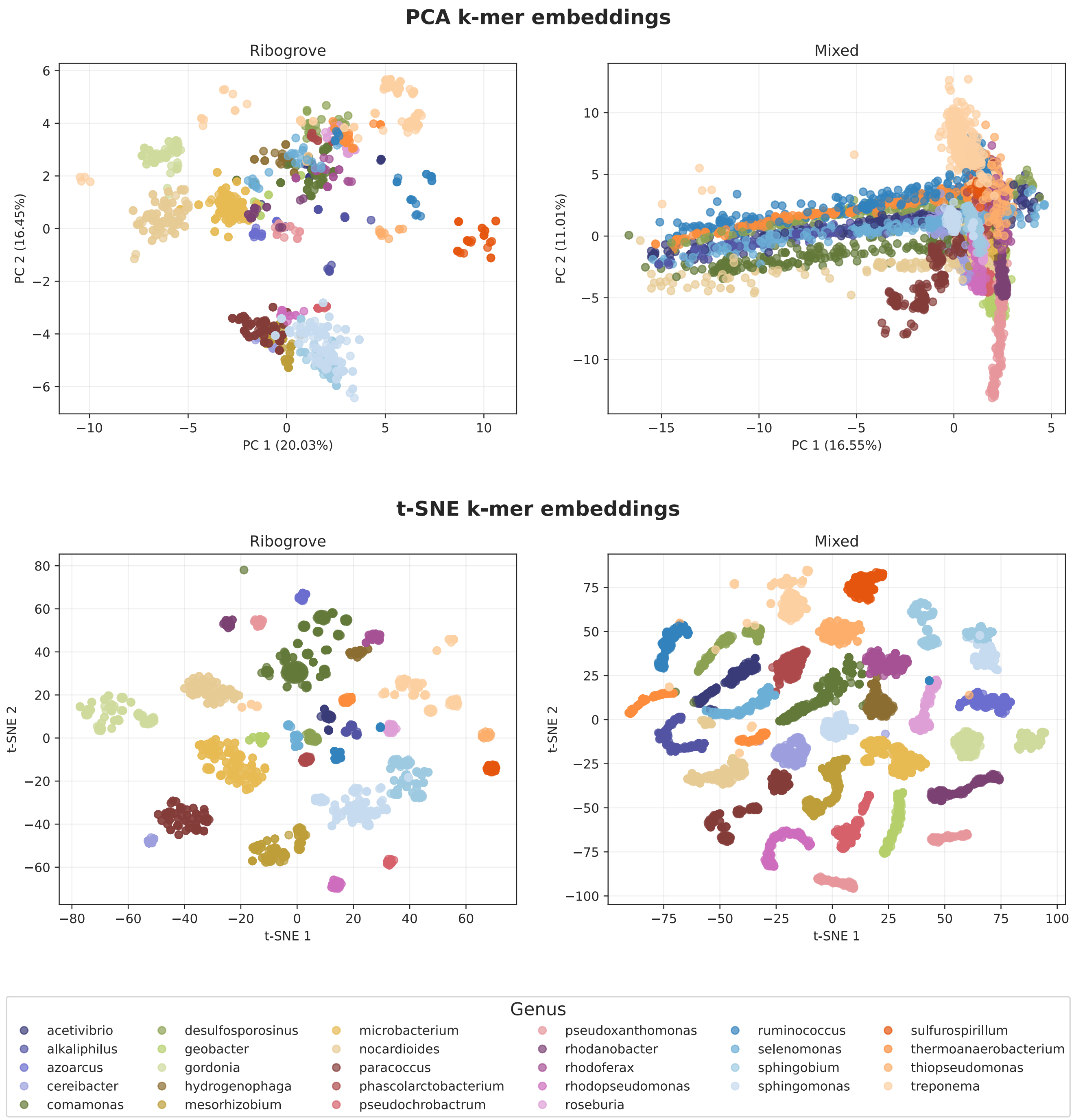

Figure S1: PCA and t-SNE of k-mer embeddings. PCA and t-SNE were employed as ordination techniques to visualize the structure of k-mer embeddings. Two datasets are shown: Ribogrove, and Mixed (combination of real and synthetic sequences). Each point represents an embedded sequence and is colored according to its genus.

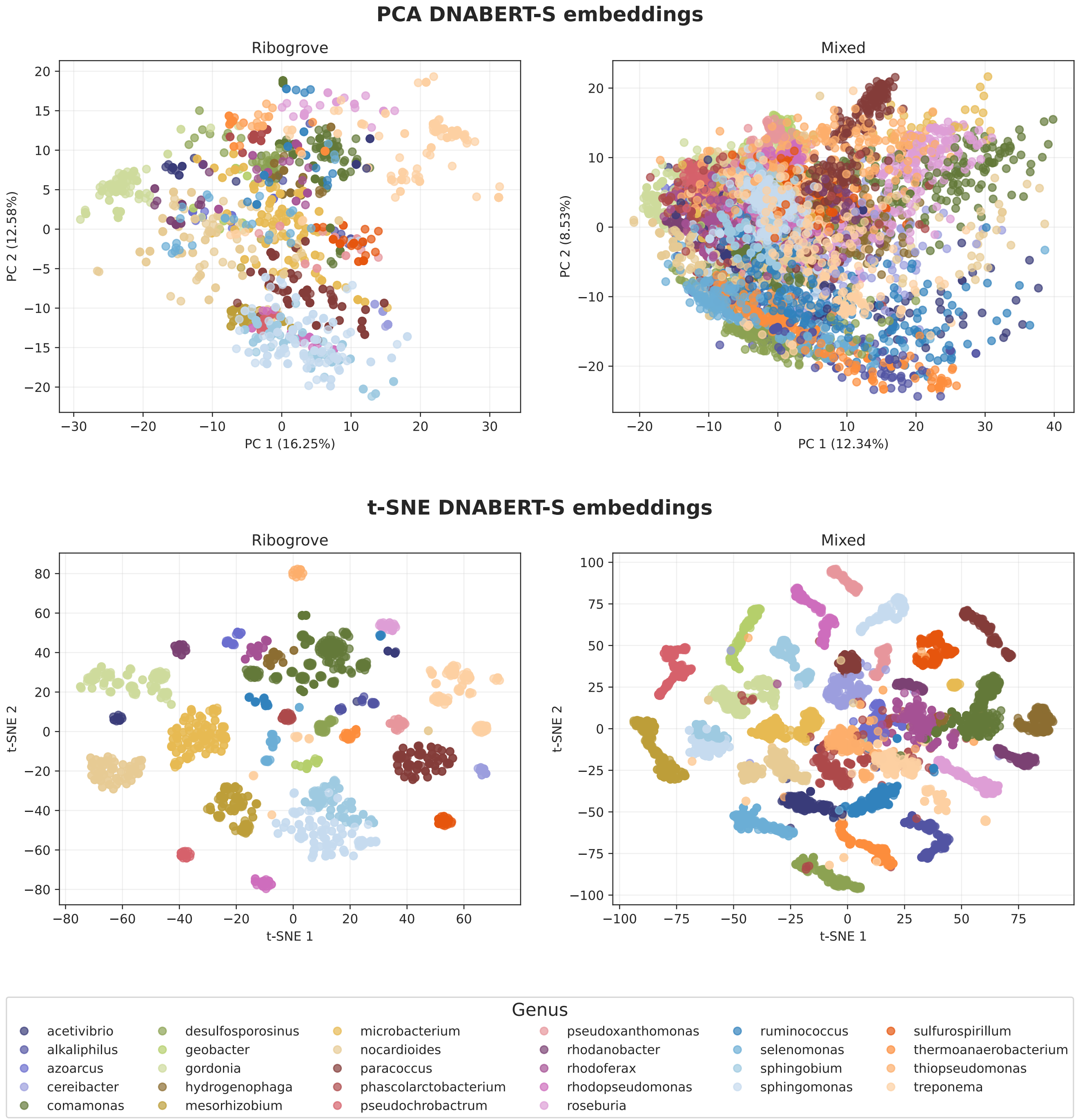

Figure S2: PCA and t-SNE of DNABERT-S embeddings. PCA and t-SNE were employed as ordination techniques to visualize the structure of DNABERT-S embeddings. Two datasets are shown: Ribogrove, and Mixed (combination of real and synthetic sequences). Each point represents an embedded sequence and is colored according to its genus.

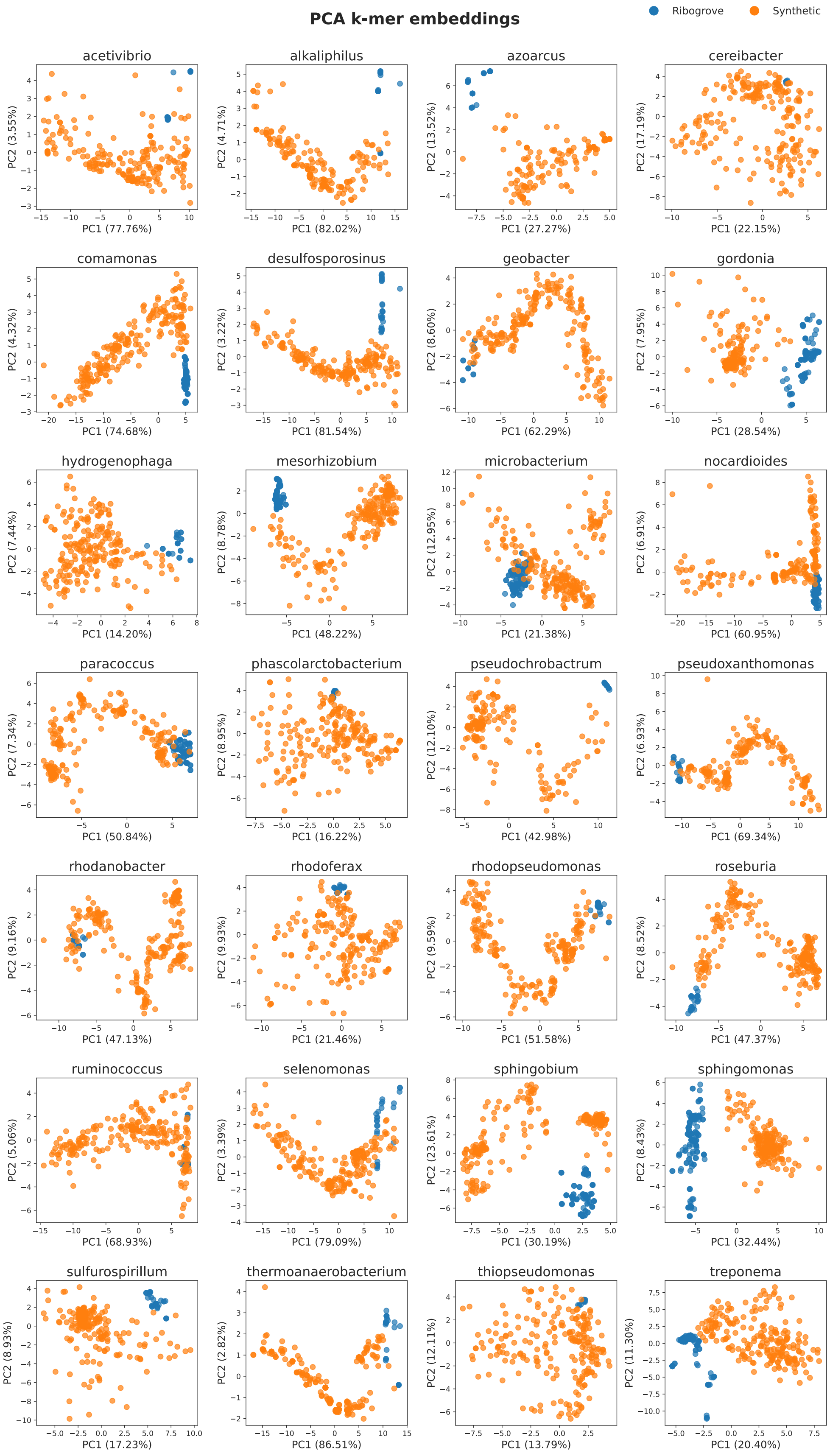

Figure S3: Per genus PCA of k-mer embeddings. Each point represents an embedded sequence and is colored by whether it is original (Ribogrove) or synthetic (generated by SGenerator).

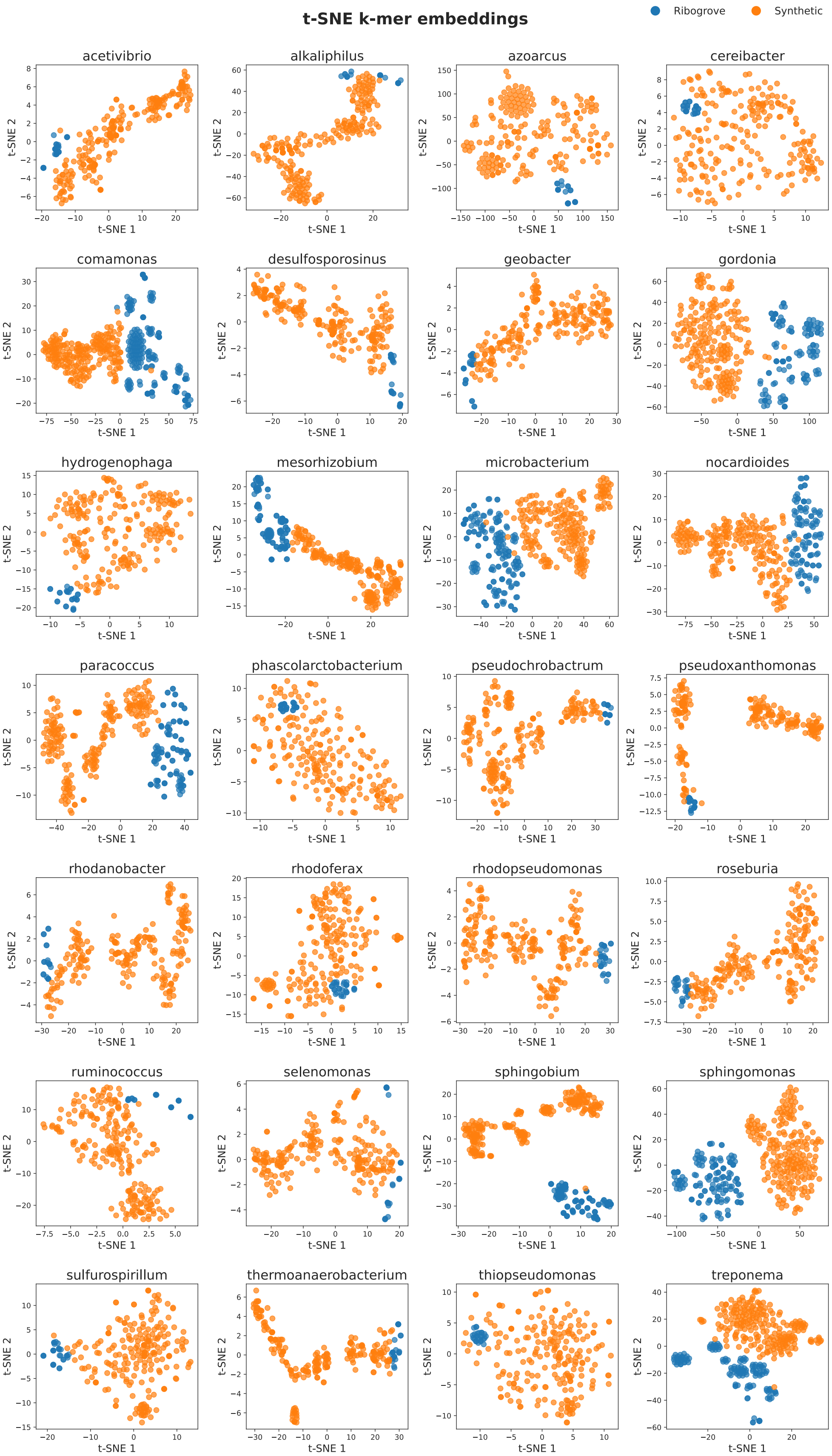

Figure S4: Per genus t-SNE of k-mer embeddings. Each point represents an embedded sequence and is colored by whether it is original (Ribogrove) or synthetic (generated by SGenerator).

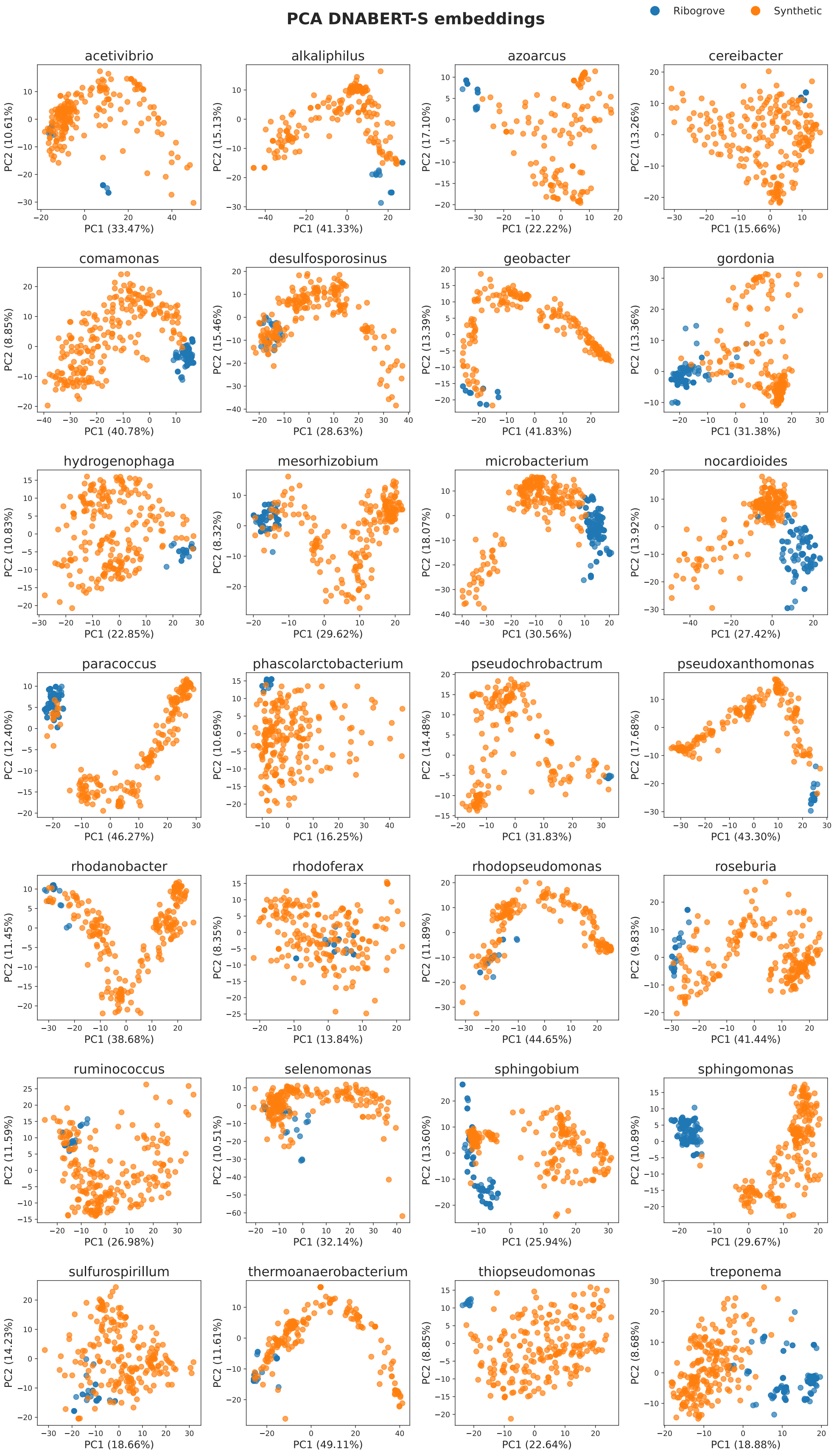

Figure S5: Per genus PCA of DNABERT-S embeddings. Each point represents an embedded sequence and is colored by whether it is original (Ribogrove) or synthetic (generated by SGenerator).

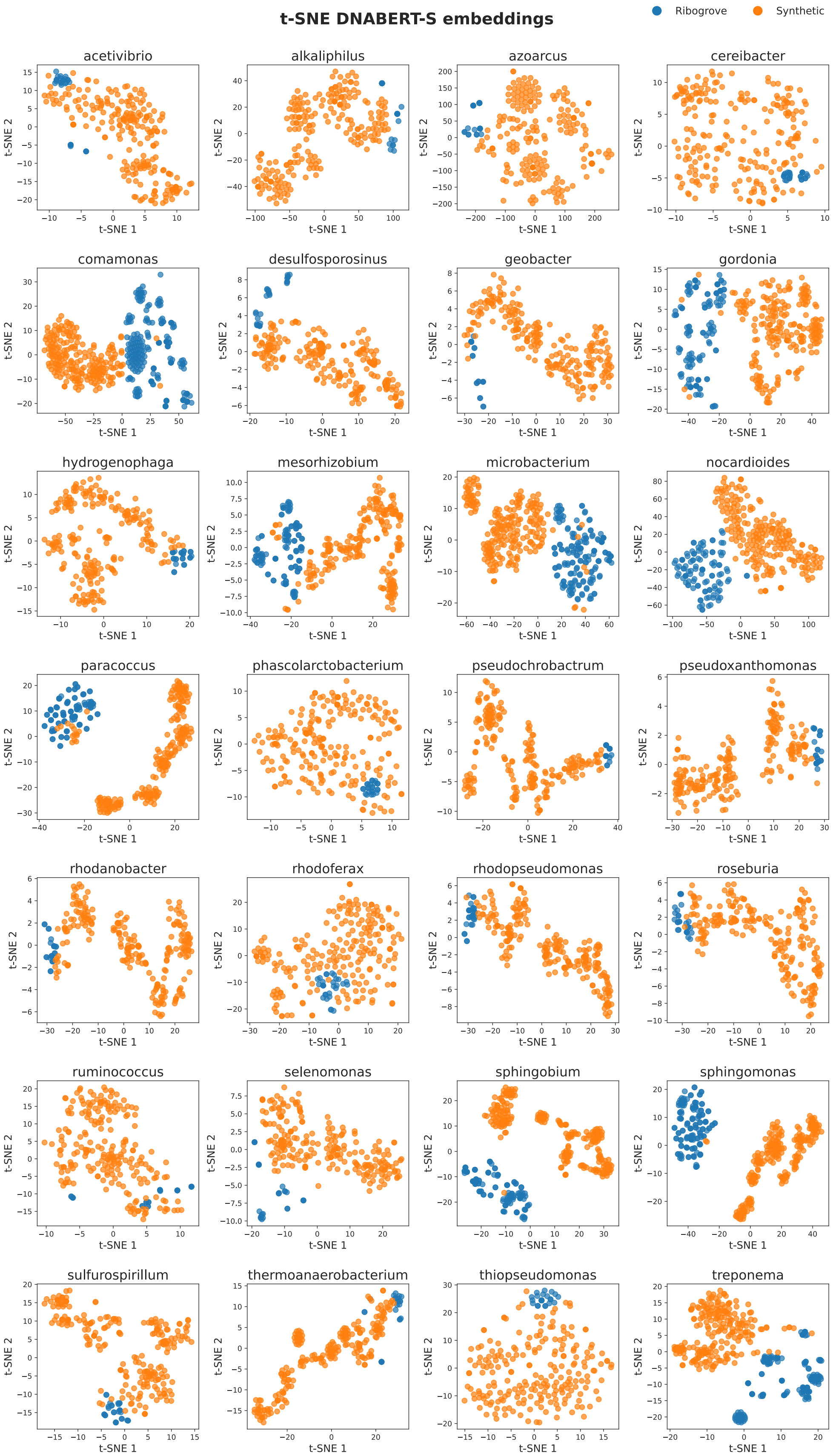

Figure S6: Per genus t-SNE of DNABERT-S embeddings. Each point represents an embedded sequence and is colored by whether it is original (Ribogrove) or synthetic (generated by SGenerator).

3 Boxplots

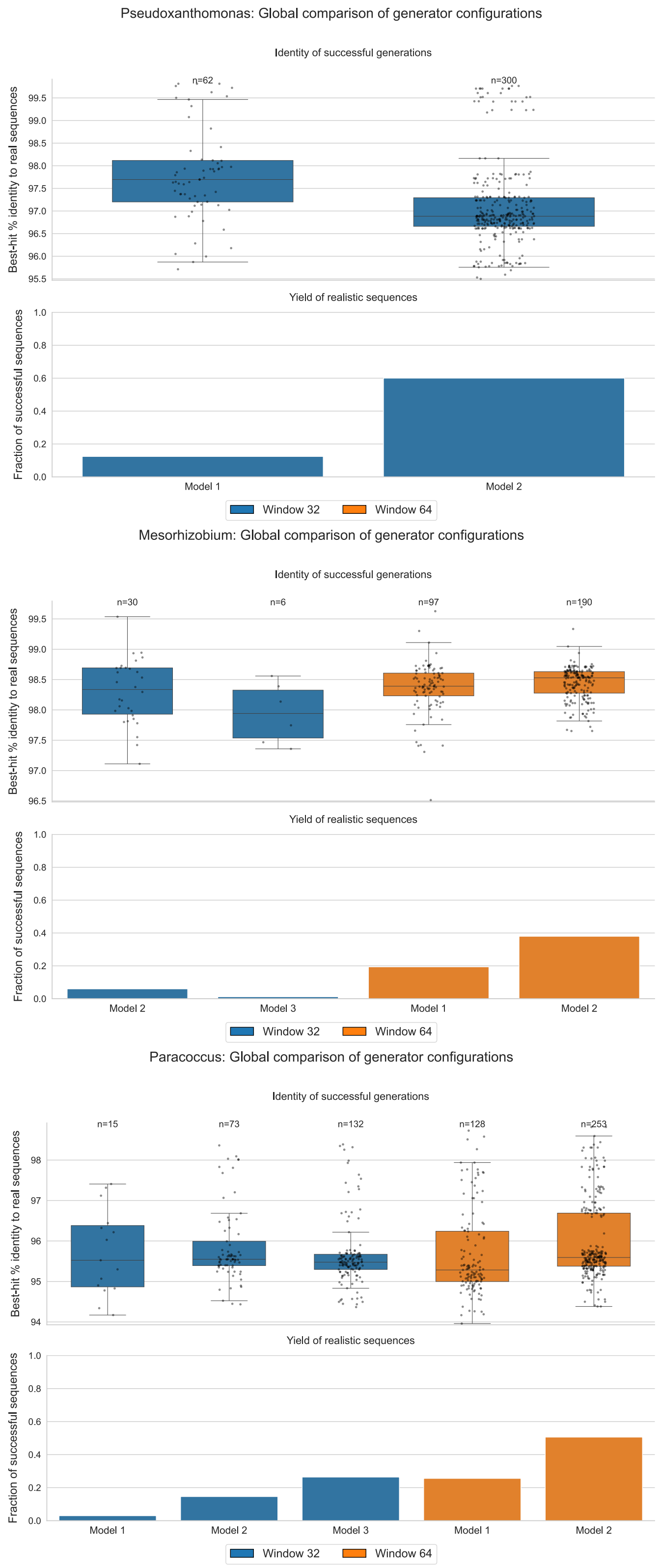

Figure S7: Additional genera: sequence quality across generator configurations. Boxplots are coloured as in Figure 3 and show the effect of model/window adjustments for generating biologically meaningful sequences.

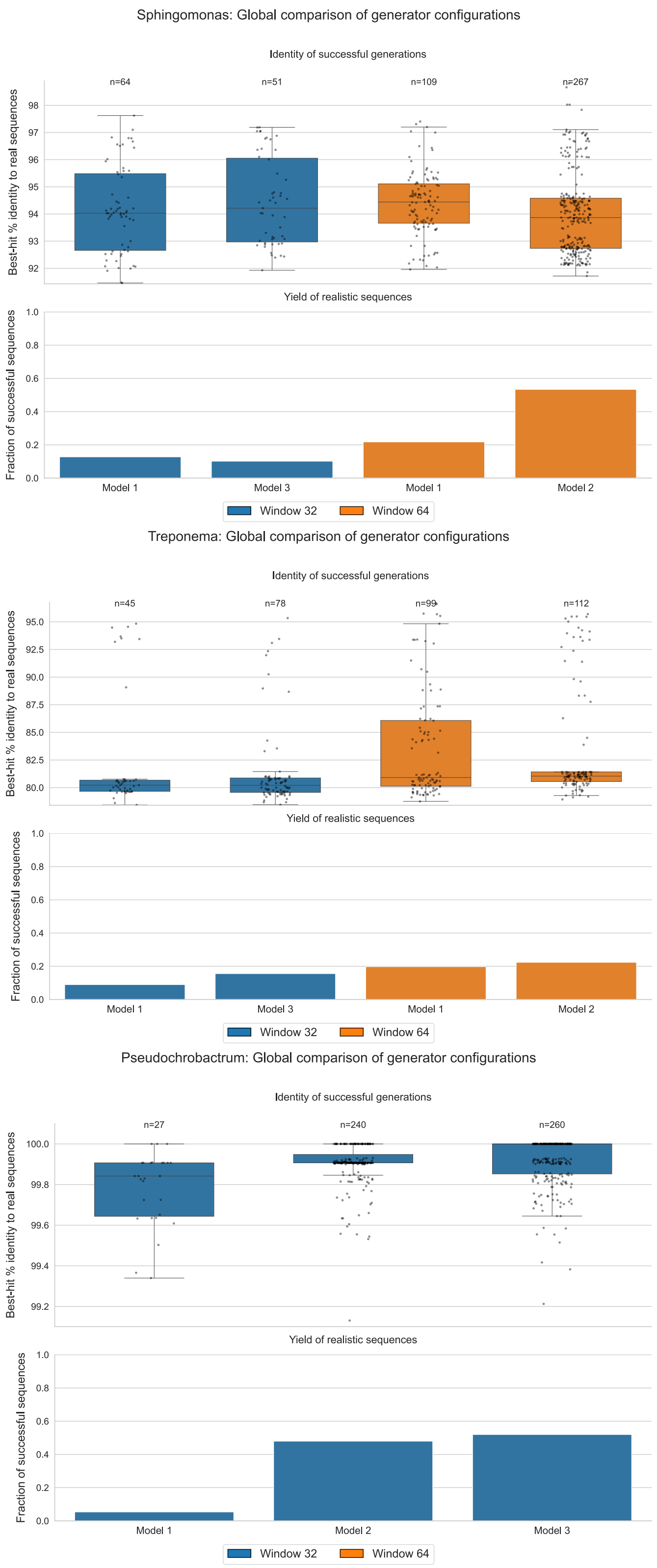

Figure S8: Additional genera (continued): sequence quality across generator configurations. Boxplots and colouring follow the conventions established in Figure 3.

4 Subtrees

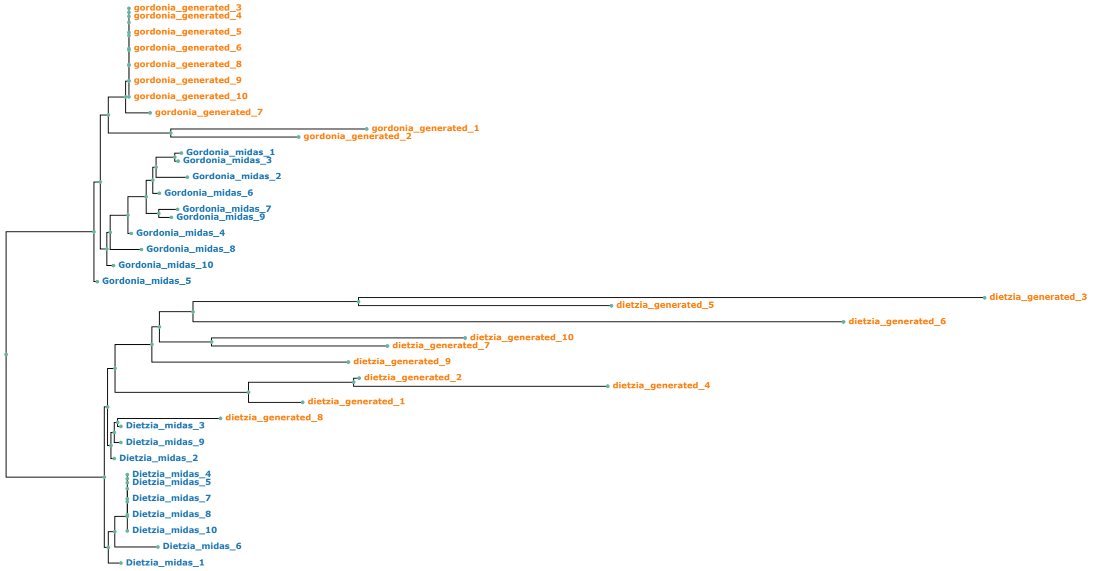

Figure S9: Phylogenetic sub-tree showing real and synthetic Gordonia and Dietzia sequences. A subsection of the full phylogenetic tree built from the MiDAS test database combined with SGenerator outputs. Real sequences are shown in blue, synthetic in orange. Despite differing training set sizes (Gordonia: 142, Dietzia: 20), synthetic sequences generated with Model 1 (window 32) cluster within the same clades as real sequences. This indicates that the model captures biologically relevant 16S rRNA variation, supporting the ecological plausibility of synthetic sequences even from limited data.

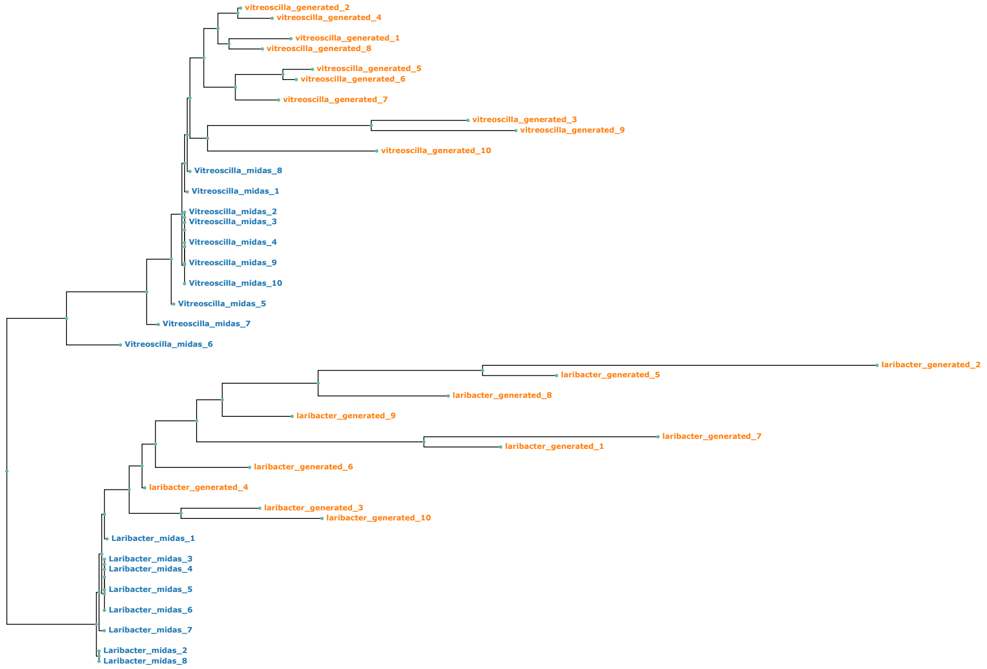

Figure S10: Phylogenetic subtree of Laribacter and Vitreoscilla. Real sequences are shown in blue and synthetic in orange. Synthetic sequences generated with Model 3 cluster closely with real Laribacter sequences despite the limited training data, indicating that the generator captures biologically meaningful 16S rRNA patterns and preserves ecological coherence even for sparsely represented taxa.

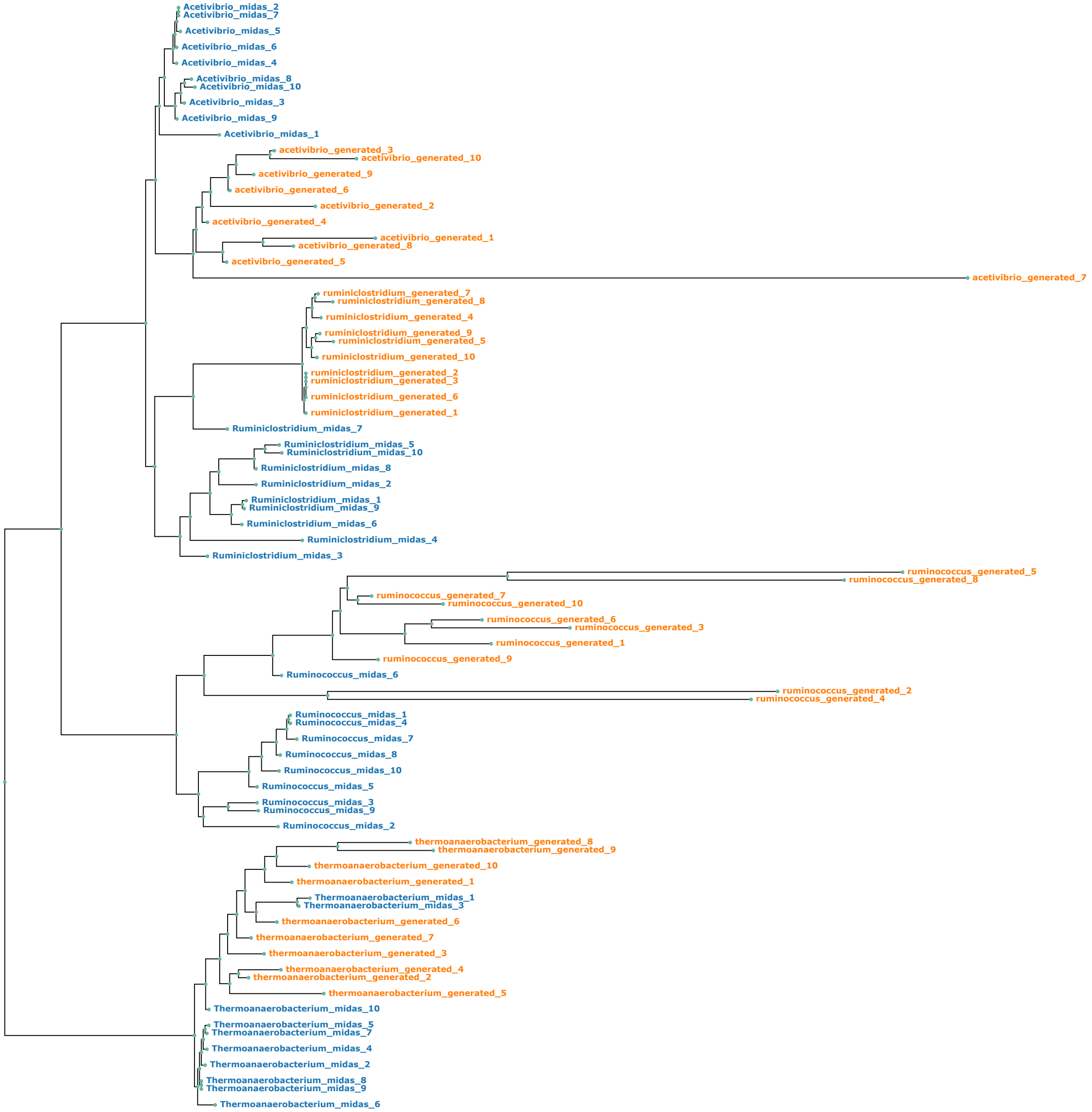

Figure S11: Phylogenetic subtree of Acetivibrio, Ruminococcus, Ruminiclostridium, and Thermoanaerobacterium. Real sequences are shown in blue and synthetic in orange. All genera were trained with similar data sizes (21–29 sequences), and synthetic sequences cluster within or alongside real sequence clades. Notably, Thermoanaerobacterium synthetic sequences generated with Model 2 fall within the main real clade, indicating improved biological coherence with the scaled architecture.

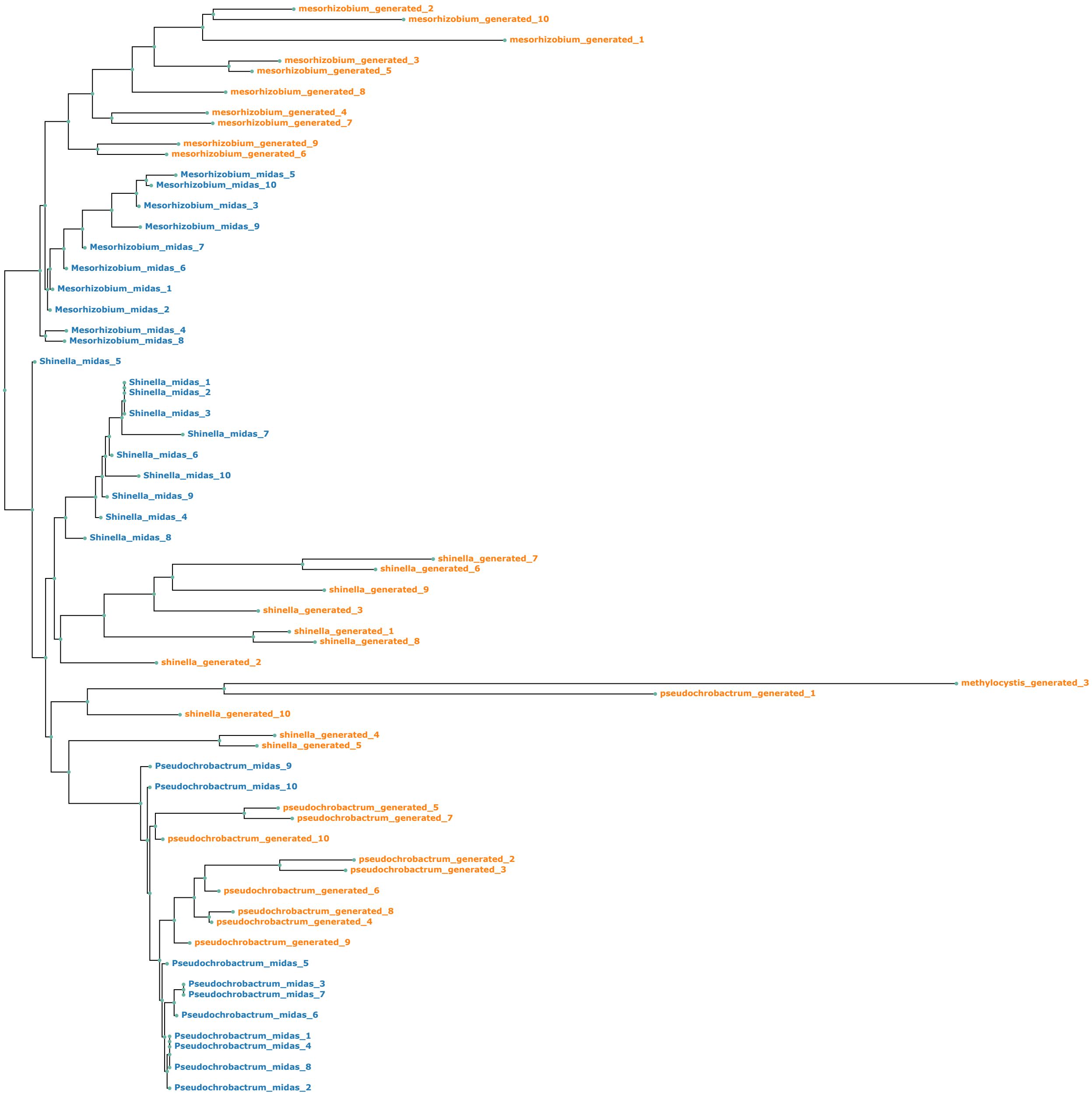

Figure S12: Phylogenetic subtree of Shinella, Methylocystis, Mesorhizobium, and Pseudochrobactrum. Real sequences are shown in blue and synthetic sequences in orange. Synthetic sequences generated for Mesorhizobium cluster into well-defined, genus-consistent clades alongside real sequences, consistent with the larger training set and scaled generator architecture. In contrast, Shinella and Pseudochrobactrum show hierarchical clustering, with synthetic sequences spanning closely related branches, reflecting limited training data and differences in model configuration. These patterns illustrate how data availability and model scaling affect the phylogenetic structure of synthetic sequences.
